# Modelling and predicting the spatio-temporal spread of cOVID-19 in Italy

**DOI:** 10.1186/s12879-020-05415-7

**Published:** 2020-09-23

**Authors:** Diego Giuliani, Maria Michela Dickson, Giuseppe Espa, Flavio Santi

**Affiliations:** 1 Department of Economics and Management, University of Trento, Via V. Inama 5, Trento, 38122 Italy; 2 Department of Economics, University of Verona, Via Cantarane 24, Verona, 37129 Italy

**Keywords:** COVID-19, Italy, Spatio-temporal model

## Abstract

**Background:**

The Severe Acute Respiratory Syndrome Coronavirus 2 (SARS-CoV-2) was first detected in China at the end of 2019 and it has since spread in few months all over the World. Italy was one of the first Western countries who faced the health emergency and is one of the countries most severely affected by the pandemic. The diffusion of Coronavirus disease 2019 (COVID-19) in Italy has followed a peculiar spatial pattern, however the attention of the scientific community has so far focussed almost exclusively on the prediction of the evolution of the disease over time.

**Methods:**

Official freely available data about the number of infected at the finest possible level of spatial areal aggregation (Italian provinces) are used to model the spatio-temporal distribution of COVID-19 infections at local level. An endemic-epidemic time-series mixed-effects generalized linear model for areal disease counts has been implemented to understand and predict spatio-temporal diffusion of the phenomenon.

**Results:**

Three subcomponents characterize the fitted model. The first describes the transmission of the illness within provinces; the second accounts for the transmission between nearby provinces; the third is related to the evolution of the disease over time. At the local level, the provinces first concerned by containment measures are those that are not affected by the effects of spatial neighbours. On the other hand, the component accounting for the spatial interaction with surrounding areas is prevalent for provinces that are strongly involved by contagions. Moreover, the proposed model provides good forecasts for the number of infections at local level while controlling for delayed reporting.

**Conclusions:**

A strong evidence is found that strict control measures implemented in some provinces efficiently break contagions and limit the spread to nearby areas. While containment policies may potentially be more effective if planned considering the peculiarities of local territories, the effective and homogeneous enforcement of control measures at national level is needed to prevent the disease control being delayed or missed as a whole. This may also apply at international level where, as it is for the European Union or the United States, the internal border checks among states have largely been abolished.

## Background

Since the first cases occurred in Wuhan (China), Coronavirus disease 2019 (COVID-19) caused by SARS-CoV-2 virus, has raised serious concerns. The virus, which made the inter-species jump to humans probably from bats through another intermediate animal host [1], causes a severe respiratory syndrome which is airborne transmitted and is characterized by a high person-to-person risk of contagion [2, 3]. Not surprisingly, SARS-CoV-2 has spread throughout and outside China in a short time following an uneven spreading pattern, despite the severe control measures put in place in the Wuhan region. For several weeks, Italy has been the second most affected country in the World and the first in Europe, with more than 70000 confirmed cases one month after the first official diagnosed case of COVID-19 [4]. The government reacted promptly by setting a quarantine zone in the areas firstly involved in the epidemic. Unfortunately, this was not enough to contain the spread of infections and hence it was also necessary to put the entire country on lockdown, which took a long period to manifest its positive effects.

The reasons of Italy’s strong involvement are not yet clearly identified, especially because it seems this was not the first European country involved in the epidemic [5]. So far, the scientific literature has suggested different concurrent explanations, such as a higher share of elderly people in the Italian population [6], a higher concentration of air pollution in the northern part of the country [7], and an overwhelming unexpected pressure on the healthcare system which has made some hospitals contributing to the transmission of the virus in the early days of the epidemic [6, 8]. Further studies will be necessary to understand the anomalously strong Italy’s COVID-19 outbreak. What is evident at the moment is that the spread of COVID-19 in Italy has not followed a uniform pattern on the territory. After the first case not directly connected with China has been discovered on 20 February 2020 in the province of Lodi (north-west Italy), the disease spread mainly throughout the northern regions of Lombardy, Veneto and Emilia-Romagna, whereas as of 31 May 2020, the 231382 cases recorded nationwide are divided mainly between Lombardy (88096 cases, 38.1% of the total), Piedmont (30496 cases, 13.2% of the total), Emilia-Romagna (27894 cases, 12.1% of the total) and Veneto (18838 cases, 8.1% of the total).

One of the elements which may have played a role is the heterogeneity amongst Italian regions, which is particularly marked between southern and northern regions, with the latter representing the industrial, economic and financial epicentre of the country [9]. Furthermore, on the northern Italian territory insists large flows of people coming from other areas because of labour commuting, and also of people looking for a more efficient healthcare, which is located right in the area where the virus has occurred with greater force [10]. These differences might have obvious influences on the spread pattern of COVID-19. Strict measures to prevent and contain the epidemic, including social distancing, closure of businesses and schools, prohibitions of travel and going outdoor, were enforced starting from the cited regions and later applied to the entire country. It is therefore of striking importance to trying to understand the contagion phenomenon and to predict its spatial diffusion as well as its temporal trend.

Many scholars are trying to give a contribution to the problem, both in open online venues and on scientific publications, debating about reproductive number, mortality distributed for age and gender and, more in general, epidemic features, without neglecting to avail of mathematical and statistical models. The main contributions use deterministic epidemiological models — see [11–13] among others —, all focussing on the time evolution of the phenomena, especially with predictive purposes. The prediction of new infected, deceased and healed is certainly essential for health policy in order to estimate the capacity of a health system to cope with the stress caused by a pandemic. Nevertheless, in the recent literature the relevance of space in the diffusion of the COVID-19 is treated only marginally [14, 15], although the importance of spatial and spatio-temporal autocorrelation in epidemiology was already highlighted in the seminal book by [16]. The authors show the effectiveness of statistical methods in analyzing the incidence of some epidemics, such as e.g. measles in Cornwall, 1969–1970, cholera in London in 1849, and tuberculosis and bronchitis in Wales, 1959–1963. In the last 20 years, spatial epidemiology has been evolved rapidly, and it has found a great response in medical applied research. — For a recent review on methods and applications in the field, see [17].

The importance of the space in the study of disease transmission, like all natural phenomena, answers to the first law of geography [18], according to which “everything is related to everything else, but near things are more related than distant things”. Reformulating this concept, it could be said that the phenomenon external to an area of interest affects what goes on inside. In light of this, we found imperative to include a component that can account for spatial dependence among areal units in the study of COVID-19 diffusion, in order to explain the strength and direction of the spreading on the territory as well as in time. This is particularly relevant in countries such as Italy or Germany in which the local health systems interested by the disease are strongly regionalized. The territorial specificity, which can result in more or less drastic containment measures, cannot be neglected in a model construction that has the ambition to explain how the contagion moves in space and time and to allow reliable spatio-temporal predictions.

The purpose of the present paper is to model and predict the number of COVID-19 infections, drawing out the effects of its spatial diffusion. Forecasts about where and when the disease will occur may be of great usefulness for public decision-makers, as they give them the time to intervene on the local public health systems.

## Methods

**Data** The Civil Protection Department of Italian Government according with the Ministry of Health, has begun to release a daily bulletin about COVID-19 infections in Italy since 26 February 2020, and made publicly available data on daily infections in Italian NUTS-3 regions (provinces).^1^

Data used in this paper come from the official GitHub repository of the Civil Protection Department on COVID-19 [4], and extend over 96 days, from 26 February to 31 May 2020. The Civil Protection Department daily updates the dataset to new infections, and revises previous records so that possible errors due to misreported or under-reported infections, when detected, are regularly corrected.

**Fig. 1:**
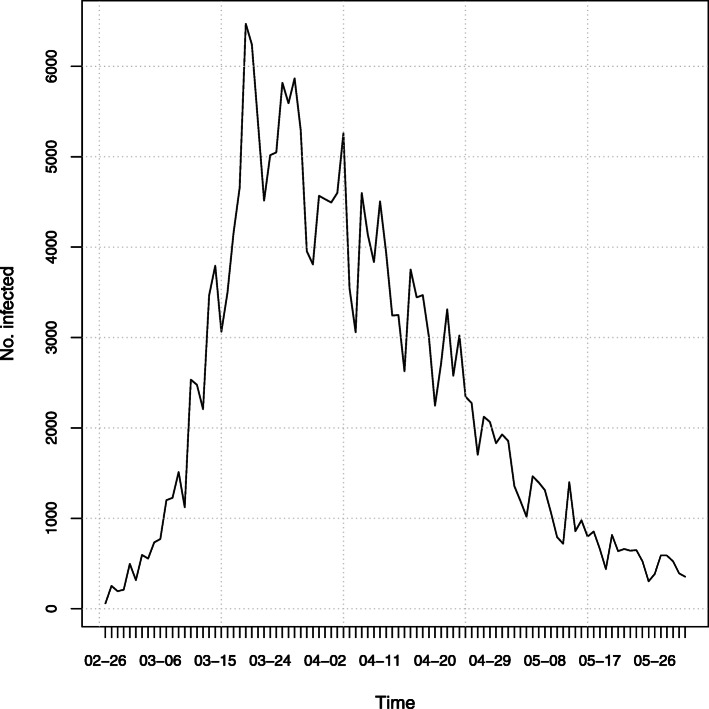
Time series of daily COVID-19 infections in Italy between 26 February 2020 and 31 May 2020, according to data released by the *Department of Civil Protection*

**Fig. 2:**
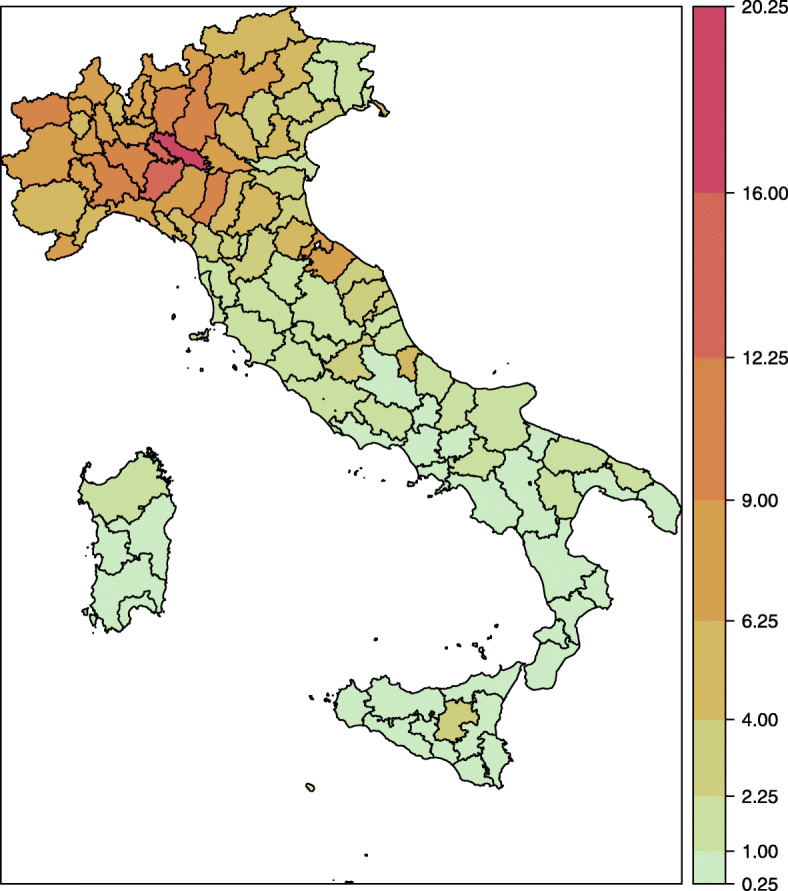
Italian provinces coloured according to cumulative COVID-19 daily incidence (total number of infections per 1000 inhabitants) in the Italian provinces (26 February 2020 – 31 May 2020). Colors toward red indicate a relatively higher incidence; colors toward green indicate a relatively lower incidence

The overall temporal evolution of daily counts of COVID-19 infections is depicted in Fig. 1,^2^ while the spatial distribution is showed in the map of Fig. 2. The first graph shows that the temporal evolution of the number of daily infections has followed an increasing trend until the end of March, which was then followed by a first slow decrease and then by a more rapid decline, according to the desired effect of control measures. In particular, the decline has regarded also the last four weeks of the series, despite the relaxation of containment measures started step-by-step from 3 May 2020. The second graph shows that the geographical distribution of the phenomenon is quite inhomogeneous, being characterized by a clear spatial pattern. (The plots of the disaggregated time series for all Italian provinces are reported in the Supplementary Material p. 3).

**Methodology** The evolution of the number of daily infections is studied by means of the count model proposed in [19] and [20], which belongs to the family of Spatial Generalised Linear Mixed Models (SGLMM). (See [21, 22] for two implementations of the model in epidemiology.)

The model treats the number of infections (denoted by *Y*_*r*,*t*_) recorded in a province (*r*) on a given day (*t*) as a realisation of a negative binomial random variable, conditionally on the infections observed in the previous periods. The adoption of the negative binomial distribution enables the variance of the number of infections to vary freely with respect to the mean, as opposed to what happens for the Poisson distribution (where they must be equal), and this allows to account for possible underdispersion or overdispersion of the distribution.

On the other hand, the mean of the distribution (*μ*_*r*,*t*_) is additively decomposed into three different terms, which describe three distinct statistical characteristics of the phenomenon under investigation: the temporal dependence (temporal autocorrelation), the geographical dependence (spatial autocorrelation) and the geographical heterogeneity. In the following, each component is briefly outlined, whereas the interest reader is referred to the Supplementary Material (p. 1 ff.) for details.

The first component catches the number of infections which are attributable to the temporal evolution of contagions within each province and depend on province-specific speed of propagation of the disease. Since the component determines the temporal dynamics of the contagion within each province, it is referred to as epidemic-within and formally is modelled by including the number of infections recorded in the same province the day before (*Y*_*r*,*t*−1_). The multiplicative coefficient (*λ*_*r*,*t*_) which determines the contribution of past infections to the expected number of infections is allowed to vary across provinces by means of a random effect.

The second component of the model accounts for the number of infections which are explained by the incidence of COVID-19 in neighbouring regions. The spread of contagion amongst provinces which are geographically close to each other substantially contributes to this term, as it is proved by the diffusion of the virus from a limited number of foci to a wider area, which interested, with unequal intensity, all the country. Formally, this component is included into the model as a weighted average of incidence rates ($Q_{r',t}\phantom {\dot {i}\!}$) of provinces ($r',r'',\dots $) which share a border with the reference region (*r*). The multiplicative coefficient (*ϕ*_*r*,*t*_) that modules the effect of neighbours’ average incidence rate on the expected number of contagions (*μ*_*r*,*t*_) is allowed to vary amongst provinces through a random effect (like in case of the temporal autoregressive coefficient *λ*_*r*,*t*_). In the following, this source of contagion is referred to as *epidemic-between* component, as it concerns the inter-province spread of COVID-19.

The last portion of cases is attributed to province-specific conditions, such as the demographic structure of population, which determined the first centres of infections and the initial exposure to the risk of contagion. We modeled this local component of the overall daily province infections as a function of time and of share of population over 65, whereas heterogeneity amongst provinces is included into the model by means of a random effect.

[20] refers to this third component as *endemic*, nevertheless, this term does not imply in this context any epidemiological qualification of the COVID-19 in the population of Italian provinces. Both terms *epidemic* and *endemic* have been inherited from [20], whereas terms *within* and *between* are introduced in this paper in order to distinguish between the temporal and spatial terms which [20] jointly refer to as *epidemic component*.

All analyses presented in this paper have been carried out using R [23] and package surveillance [24] in particular.

## Results

The estimated model provides useful insights about the evolution and spread of COVID-19 occurrences across Italian provinces. The most striking evidence is that the epidemic potential across areas is, on average, very strong; however, it is also highly heterogeneous among provinces.

**Fig. 3:**
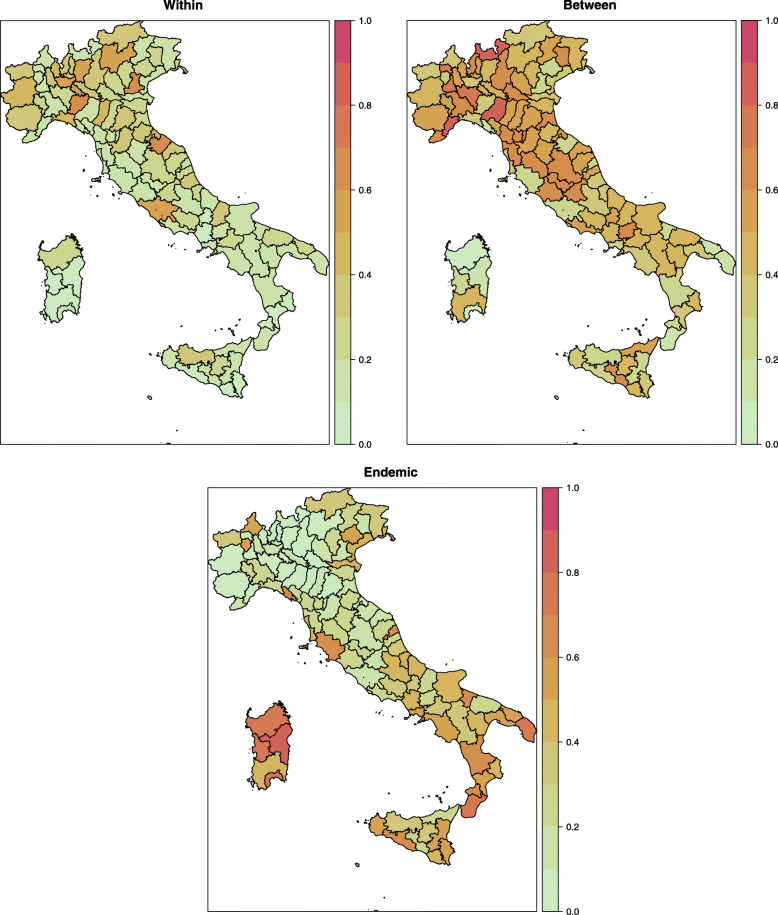
Maps of the three fitted mean number of infections components, averaged over all days between 26 February 2020 and 31 May 2020, by province. The values of components are represented as proportions of the total mean in order to show the relative importance of each term. Colors toward red indicate a relatively high role of the component; colors toward green indicate a relatively low role of the component

To better assess the degree of spatial heterogeneity, Fig. 3 shows three maps depicting the composition of the estimated expected number of infections in terms of the within-epidemic, between-epidemic and endemic component. For each province, the three fitted components are expressed as proportions of their sum.

It can be clearly seen that only few provinces, the most affected by the disease until now, are mainly influenced by the local endogenous transmission of the contagion (map on the left). For a relatively higher number of provinces, mostly located in the north and centre parts of the country, a relevant number of cases is instead explained by the transmission from neighbouring provinces (map in the centre). For the majority of provinces, located mainly in the south, the contagions follow essentially the endemic trend (map on the right). The comparison among provinces in terms of the three investigated components is robust to spuriousness because the Italian population is entirely and homogeneously susceptible, being immunologically “naïve” to this new virus. Moreover, being vaccines still missing, there are no differences among provinces deriving from vaccination coverage. There could be, however, other structural differences due to, for example, an uneven territorial distribution of medical sources, and this is one of the reasons why the province-level random effects has been included into the model.

**Fig. 4:**
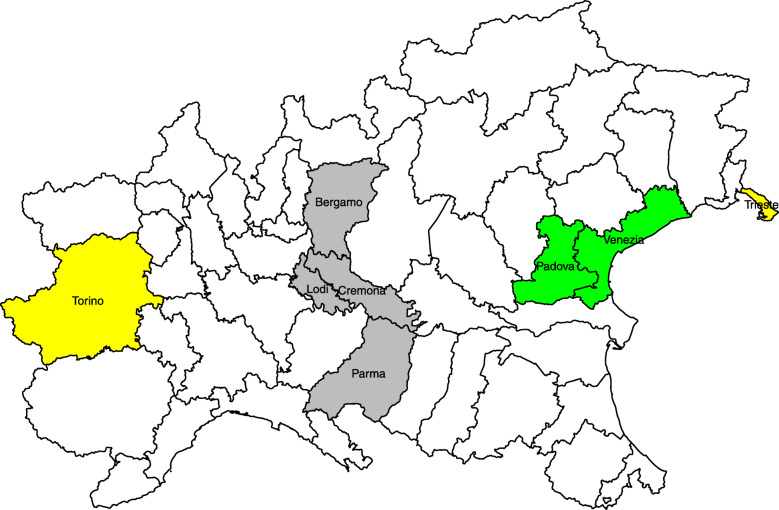
Locations of the paradigmatic provinces in the north of Italy, namely Torino (with a population density of 331 residents per square kilometer), Bergamo (404 residents/km^2^), Lodi (294 residents/km^2^), Cremona (203 residents/km^2^), Parma (131 residents/km^2^), Padova (437 residents/km^2^), Venezia (345 residents/km^2^) and Trieste (1103 residents/km^2^). These provinces cover a total area of 20411 square kilometers

**Fig. 5:**
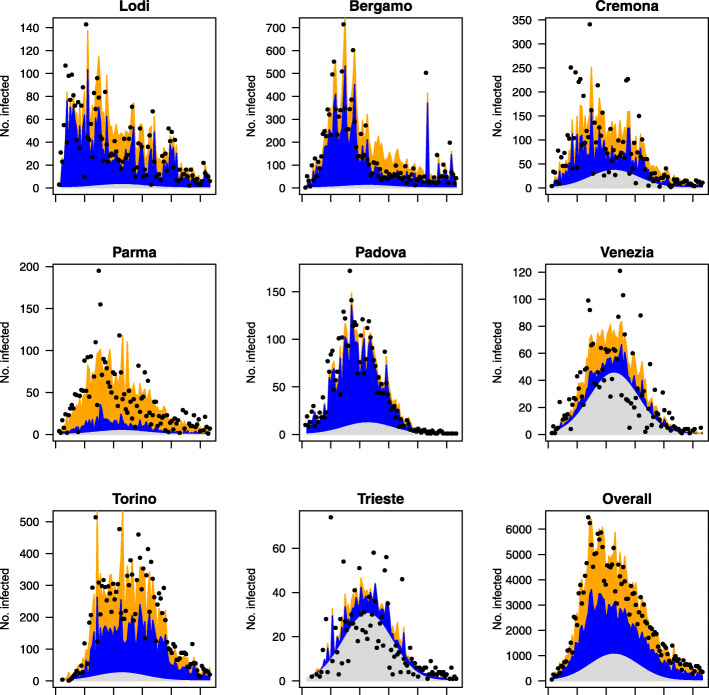
Fitted means of the three submodels for the selected provinces between 26 February 2020 and 31 May 2020. The vertical axis represents the daily number of infections and the horizontal axis represents the time in days. The dots represent the observed daily counts. The blue area represents the within-epidemic component. The orange area represents the between-epidemic component. The gray area represents the endemic component

To gain further insights about the relative importance of the three components, we take into consideration some paradigmatic provinces located in the part of the country with the highest number of cases, that is the northern areas (see Fig. 4). In particular, we focus the attention on the northern part of Italy because after the illness started to occur in the country, it took several days to spread throughout the territory, thus leading to an unbalanced spatial distribution of infections. Figure 5 depicts the mean number of cases estimated by the model along with the observed number of cases for the paradigmatic provinces. First of all, the province of Lodi (Lombardy region), which is the first area that reported cases, sees a predominance of the component related to the internal diffusion of the disease. This evidence agrees with the quarantine to which some of its municipalities underwent since the beginning of the outbreak and with the hypothesis that the epidemic in Italy started right there. The province of Lodi shares the eastern border with the province of Cremona (Lombardy region), that in turn shares the northern border with the province of Bergamo (Lombardy region), as depicted in Fig. 4. The latter two are so far some of the most severely affected provinces in Italy. Their proximity with the province of Lodi makes them widely susceptible to the contagion effect between neighbouring regions. This suggests that the infections among inhabitants occurred before containment measures were carried out and that the disease remained undetected before patient 1 was discovered. However, between the start of the national lockdown (on 11 March) and 31 May, both in province of Cremona and in province of Bergamo, the expected number of infections due to the between-epidemic component has decreased by 66%. A similar situation arose for the province of Parma (Emilia-Romagna region), which share the northern border with the province of Cremona. Even here the between-epidemic component is relevant and it may be explained with the strong social and economic ties existing among the areas. The second in time discovered outbreak of COVID-19 in Italy was detected in a small town in the province of Padova (Veneto region). Even in this case, strong control measures to reduce transmission outside have been carried out, as evidenced by the almost total influence of the within-epidemic component. This shows that there have been no “return infections” from the surrounding areas but it does not mean that, before the quarantine, the infection could not have circulated in the neighbouring provinces. In fact, the province of Venice (Veneto region), which is geographically, culturally and economically near to that of Padova demonstrates to be affected by these form of interactions. The cases of the provinces near the national borders are also of particular interest, such as the province of Trieste (Friuli Venezia Giulia region), adjoining with Slovenia. This province may be affected by the so-called boundary problem for which the analysis of a phenomenon may be biased by the presence of arbitrary administrative geographic borders. Thus, on one hand, we might lose information regarding what happens beyond the country border, due to different ways of collect information and monitor the diffusion of the disease. One the other hand, we might not have a clear overview about cross-border phenomena as job trajectories. In addition, this province seems to be not connected with the aforementioned outbreaks originated in Lombardy and Veneto. Due to these reasons, the province of Trieste shows a limited effect of epidemic components and a prevalence of the endemic component, which is only due to the general trend of the disease and the specific characteristics of this territory, such as the overwhelmed local medical sources or the population structure. Therefore, if we focus on the country as a whole, and hence on the national aggregated counts, the contribution of the three components seems quite balanced. For the individual provinces, instead, they can exert a very different role.

Moreover, to check the ability of the proposed model in explaining the spatio-temporal distribution of the COVID-19 occurrences in Italy, we examine how it is good at predicting the future daily counts of infections. Therefore, we re-estimate the model using the data between 26 February 2020 – 30 May 2020 and we make prediction for 31 May 2020. The observed total number of cases at 31 May 2020, obtained by aggregating the province data, is 354; the model provides a prediction of 358 cases. Therefore, the model overestimates by only 4 units. Naturally, the advantage of the proposed model is that it provides predictions for each individual province (point predictions can found in Tables 2 and 3 of the Supplementary Material ) but it is clear that the use of predictions at local level can have positive effects on the prediction of total number at country level. We report (in Fig. 2 of the Supplementary Material ) the 95% prediction intervals of the provincial counts of infections at 31 May 2020. Despite the fact that the intervals are quite wide, the results are promising if compared with the known observed counts. Indeed, the root mean squared prediction error and median absolute prediction error of provincial counts are, respectively, 51.139 and 239.453. We expect that the level of precision will improve as the data are updated and the observed time series become longer.

## Discussion

In this article, we analyzed, by modeling, the trend of COVID-19 epidemics along time and space. The use of spatio-temporal models can greatly improve the estimation of the number of infected and can help the public decision-makers to better plan health policy interventions. Italy has viewed a massive spread of the disease with peculiar patterns on the territory. Started from some provinces in the northern area, this serious illness descended down the Boot and is nowadays present in all 107 Italian provinces. Containment measures in Italy have followed an application in three steps: first, some municipalities in Lombardy and Veneto regions underwent to quarantine; second, the entire Lombardy and some provinces in other northern regions (Veneto, Piedmont, Emilia-Romagna and Marche) were isolated from the rest of the country; third, all Italian territories were subjected to a complete lockdown. Such a stepwise approach in imposing hard control measures to the entire national territory might have conducted to a temporal shifting of the contagion dynamics.

Emblematic is the case of the province of Lodi, the first area submitted to the quarantine. In that province, the number of recorded daily infections reached 85 when the illness began to be systematically discovered, it settled the average value of 30 cases per day one month after the lockdown of the area (thus the new detected contagions have been more than halved), and finally it dropped to 9 cases per day on 31 May. These numbers suggested that policies of contagion containment exert a mild success in the areas where there was an effective enforcement of the control measures. This is the case of the province of Milan, which faced a large number of cases and is still not experiencing a final and decisive reduction in the number of new infections.

Central and south provinces have viewed a delayed start of the epidemics but also the arrival, in the last two weeks, of flows of people escaped from northern regions undergone to lockdown. As an example, the province of Florence went from an average increment of 3 new cases in the first period to an average increment of 110 cases after one month and to 5 cases at the end of the study period. Similarly, the province of Naples has gone from an average increment of 4 new cases, to an average increment of 96 new cases after one month, and to 3 cases at the end of May. This evidence confirms the hypothesis of downhill race of the disease along the peninsula and that the experience of quarantine in the north of Italy has avoided its spread in centre-south Italy.

These differences in the dynamics of the epidemics in Italy demonstrate the crucial importance of a strong national coordination level for the homogeneous enforcement of control measures, but also reveal how essential predictions at local level are. Since the epidemics started, a frequent question of the public decision-maker concerned when the peak of contagions will manifest. As our findings suggest, a more appropriate question should have dealt with the emergence of different local peaks in different moments, since the very heterogeneous shape of the illness diffusion. The dramatic events in northern provinces served as a test bench for the health system, offering an overview about what southern provinces might be confronted and probably other European countries that share with Italy similar systems. Analyses and predictions both in space and time offer a decisive perspective about which areas may be more affected and when, given the time to the central decision-makers to intervene on the local policies.

## Conclusions

In this article the trend of COVID-19 epidemics has been analyzed along time and space. The use of spatio-temporal models at provincial level greatly improves the predictions of the number of infected people and can help the public decision-makers to better plan health policy interventions.

Italy has viewed a massive spread of the disease with peculiar patterns on the territory. Started from some provinces in the northern area, this serious illness descended down the Boot and is nowadays present in all 107 Italian provinces. Differences emerged in the dynamics of the epidemics in Italy demonstrate the crucial importance of a strong national coordination level for the homogeneous enforcement of control measures, but also reveal how essential predictions at local level are. Since the epidemics started, a frequent question of the public decision-maker is about when the peak of contagions will manifest. Probably a more appropriate request should concern the emergence of different local peaks in different moments, which Italy should expect in next weeks. The dramatic events in northern provinces can serve as a test bench for the health system, offering an overview about what southern provinces might be confronted and probably other European countries that share with Italy similar systems. Analyses and predictions both in space and time offer a decisive perspective about which areas may be more affected and when, given the time to the decision-makers to intervene on the local policies.

## Supplementary information

**Additional file 1** **Supplemental information 1** Methodological details, and further results.

## Data Availability

The datasets used and/or analysed during the current study are available from the corresponding author on reasonable request.

## Abbreviations

SARS-CoV-2: Severe acute respiratory syndrome coronavirus 2
COVID-19: Coronavirus disease 2019
NUTS: Nomenclature of territorial units for statistics
SGLMM: Spatial generalised linear mixed models

## Footnotes

1 Acronym “NUTS” stands for *Nomenclature of Territorial Units for Statistics*, and refers to the hierarchical classification of European regions (https://ec.europa.eu/eurostat/web/nuts/background) as defined by the European Commission. The NUTS classification is based on four nested levels of aggregation: sovereign countries are referred to as NUTS-0 regions, whereas the smallest territorial units are NUTS-3 regions.

2 The overall time series here depicted is given by the aggregation of regional time series and may not correspond exactly to the national time series because some cases could not being assigned to the province where they occurred. Anyway, the difference between the two series is negligible.
