# Supplementary material for: Modelling and predicting the spatio-temporal spread of cOVID-19 in Italy
Source: BMC Infect Dis. 2020 Sep 23;20:700. doi: 10.1186/s12879-020-05415-7 (PMC7509829; doi:10.1186/s12879-020-05415-7)
Supplement: Supplementary file 1 — Additional file 1 Supplemental information 1 Methodological details, and further results. [file 12879_2020_5415_MOESM1_ESM.pdf]

Modelling and predicting the spatio-temporal spread of COVID-19 in Italy

Diego Giuliani[1], Maria Michela Dickson[1], Giuseppe Espa[1], and Flavio Santi[2]

[1]Department of Economics and Management, University of Trento
[2]Department of Economics, University of Verona

Supplementary Material

1 Statistical model

The data about the spatio-temporal distribution of Coronavirus disease 2019 (COVID-19) infections at province level consist in multivariate count time series whose spatial references are in the form of irregular spatial lattices. Therefore, the proper regression modelling framework for this empirical circumstance is the class of the so-called areal Generalized Linear Models (GLMs). By extending the seminal model originally introduced by Held et al. (2005), Paul and Held (2011) proposed an endemic-epidemic multivariate time-series mixed-effects GLM for areal disease counts, which proved to provide good predictions of infectious diseases (see Adegboye and Adegboye, 2017; Cheng et al., 2016, among the others).

The main equation of the model describes the expected number of infections $\mu_{r,t}$ in a region (province) r at time (day) t as follows:

$$\mu_{r,t} = \lambda_r \, Y_{r,t-1} + \phi_r \sum_{r' \neq r} w_{r',r} \, Q_{r',t-1} + e_r \, \nu_{r,t} \,, \tag{1}$$

where $Y_{r,t}$ is the number of infections reported in the region r at time t, which follows a negative binomial distribution with region-level overdispersion parameter ψ_r. If $\psi_r > 0$ the conditional variance of $Y_{r,t-1}$ is $\mu_{r,t}(1 + \psi_r \, \mu_{r,t})$, while if $\psi_r = 0$ the negative binomial distribution reduces to a Poisson distribution with parameter $\mu_{r,t}$.

The three terms on the right-hand side of Equation (1) correspond to the three components of the model: the *epidemic-within*, the *epidemic-between*, and the *endemic*.

The first component models the contribution of temporal dynamics of contagions to the expected number of infections within region r. The term includes the number of infections observed in the previous day (time $t-1$), which affect $\mu_{r,t}$ depending on the value of the coefficient $\lambda_r > 0$. As the notation suggests, λ_r changes amongst the provinces because of a random effect which allows for heterogeneous behaviour in the dynamics of contagions.

The epidemic-between component models the contagion between neighbouring provinces by including the average incidence of the infections $Q_{r',t-1}$ of provinces r' which are neighbours of province r. In particular, the coefficients $w_{r',r}$ in the summation $\sum_{r' \neq r} w_{r',r} \, Q_{r',t-1}$ are positive if either province r' and r share a border or province r' and r share a border with the same province, whereas $w_{r',r}$ is zero otherwise. The coefficient ϕ_r determines the magnitude of the effect of inter-province spread of contagion, and changes amongst provinces according to the population as well as to unobserved heterogeneity in the diffusion process.

The last component determine the province-specific contribution to the number of infections, once the temporal and spatial autoregressive effect are accounted for. The term e_r is the population of province r, whereas term $\nu_{r,t}$ consists of a national time trend component, and a province-specific effect depending on the share of population over 65, and on a random effect which catches the heterogeneity due to unobserved factors.

Paul and Held (2011) suggested that the endemic and epidemic subcomponents can be modelled themselves through log-linear specifications:

$$\log(\lambda_{r,t}) = \alpha_r^{(\lambda)} + {\boldsymbol{\beta}^{(\lambda)}}^\top \boldsymbol{z}_{r,t}^{(\lambda)} \,, \tag{2}$$

$$\log(\phi_{r,t}) = \alpha_r^{(\phi)} + {\boldsymbol{\beta}^{(\phi)}}^\top \boldsymbol{z}_{r,t}^{(\phi)} \,, \tag{3}$$

$$\log(\nu_{r,t}) = \alpha_r^{(\nu)} + {\boldsymbol{\beta}^{(\nu)}}^\top \boldsymbol{z}_{r,t}^{(\nu)} \,. \tag{4}$$

where the $\alpha_r^{(\cdot)}$ parameters are region-specific intercepts and $\boldsymbol{z}_{r,t}^{(\cdot)}$ represent observed covariates that can affect both the endemic and epidemic occurrences of infections. The varying intercepts allow to control for unobserved heterogeneity in the disease incidence levels across regions due, for example, to under-reporting of actual infections (Paul and Held, 2011). Given the regionally decentralized health system in Italy, non-negligible differences in case reporting of COVID-19 infections among Italian regions are very likely, which make the opportunity to have region-specific intercepts very important. Following Paul and Held (2011) region-specific intercepts can be obtained through the inclusion of random effects. In particular, we assume here that

$$\alpha_r^{(\lambda)} \overset{iid}{\sim} N(\alpha^{(\lambda)}, \sigma_\lambda^2), \text{ and } \quad \alpha_r^{(\phi)} \overset{iid}{\sim} N(\alpha^{(\phi)}, \sigma_\phi^2), \quad \alpha_r^{(\nu)} \overset{iid}{\sim} N(\alpha^{(\nu)}, \sigma_\nu^2).$$

The Paul and Held (2011) model with normally distributed random effects can be estimated through penalized likelihood approaches that have been implemented in the R package surveillance (Meyer et al., 2017). See Paul and Held (2011) for futher details.

1.1 Epidemic-within submodel

Given the brevity of the observed time series, the epidemic-within autoregressive parameter is assumed to be constant over time and, in absence of useful exogenous covariates, the model of Equation (2) takes the form

$$\log(\lambda_r) = \alpha_r^{(\lambda)},$$

that is, the "internal" infectiousness depends only on a spatially varying intercept.

1.2 Epidemic-between submodel

Following Meyer and Held (2014), the subcomponent model for the epidemic within autoregressive parameter, Equation (3), is here specified as

$$\log(\phi_{r,t}) = \alpha_r^{(\phi)} + \beta^{(\phi)} \log e_r,$$

which accounts for the fact that the regions may have different propensities to be affected by the other neighbouring regions, and this may depend by their resident population share. The rationale is that the more populated regions tend to be more susceptible to transmission across regions.

1.3 Endemic submodel

Since some first recent empirical evidences suggest that the number of COVID-19 infections seems to grow exponentially over time (Liu et al., 2020; Maier and Brockmann, 2020), the endemic component model assessing the temporal dynamic of disease incidence, Equation (4), is specified as a second-order polynomial log-linear regression:[1]

$$\log(\nu_{r,t}) = \alpha_r^{(\nu)} + \beta_1^{(\nu)} t + \beta_2^{(\nu)} t^2 + \beta_3^{(\nu)} \log(a_r),$$

where $t = 1, 2, \dots$ is the time in days and a_r is the proportion of inhabitants over 65 years old.

In the global model of Equation (1) the endemic predictor $\nu_{r,t}$ is multiplied by the offset e_r, which in our case is the regional share of resident population.

2 Supplementary Results

Figure 1: Time series of daily COVID-19 infections in the Italian provinces between 26 February 2020 and 31 May 2020, according to data released by the *Department of Civil Protection*. Note the sharp increase of daily infections in some northern provinces (such as, e.g., Milano, Bergamo, and Brescia) as opposed to several southern provinces (such as, e.g., Trapani and Caltanissetta) where the first infections appeared only recently and the overall number remained low.

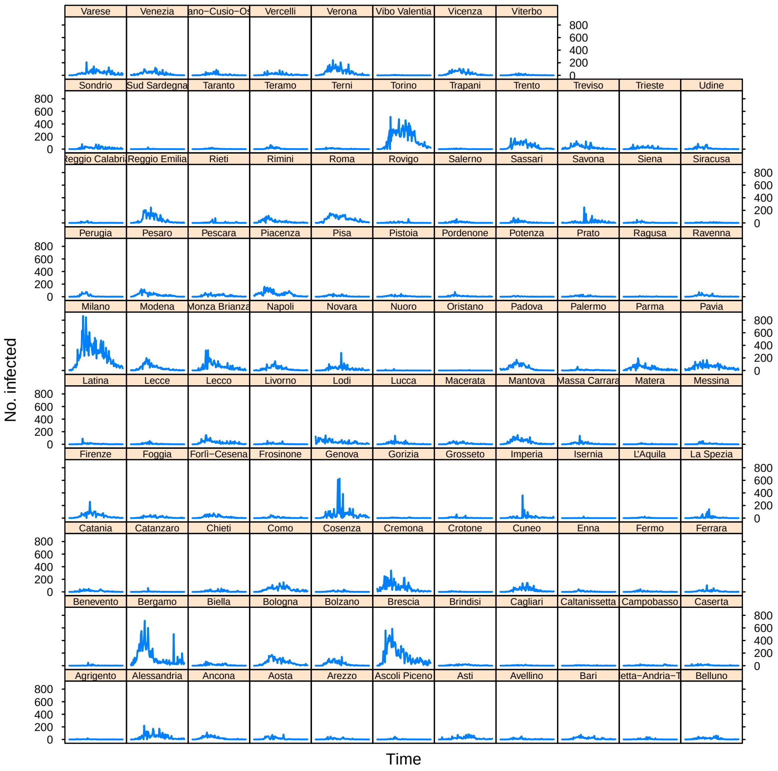

[1]We do not include higher-order terms to avoid the risk of introducing spurious endemic patterns and overfitting.

Table 1: Maximum penalized likelihood estimates of parameters of model (1). Both point estimates (second column) and standard errors (third column) refer to quantities in the first colum. Standard errors of variances of random effects (σ^2_λ, σ^2_ϕ, σ^2_ν) cannot be estimated.

Parameter	Estimate	St. Error
$\exp(\alpha_r^{(\lambda)})$	0.268***	0.021
$\beta^{(\phi)}$	0.893***	0.125
$\exp(\alpha_r^{(\phi)})$	124.0	77.940
$\exp(\alpha_r^{(\nu)})$	1941.0	2922.0
$\exp(\beta_1^{(\nu)})$	1.184***	0.008
$\exp(\beta_2^{(\nu)})$	0.998***	0.000
$\beta_3^{(\nu)}$	3.460***	1.253
σ^2_λ	0.413	–
σ^2_ϕ	0.655	–
σ^2_ν	1.001	–

***p-value < 0.01, **p-value < 0.05, *p-value < 0.1

Figure 2: Prediction intervals (confidence: 95%) of one-day ahead forecast of the number of COVID-19 infections on 31 May for all 107 Italian provinces. The number of infections is predicted according to model (1) fitted on data between 27 February 2020 and 30 May 2020. Each province is identified by the acronym (see also Tables 2 and 3), whereas prediction intervals are delimited by black interval bars. Black and red dots respectively represent point predictions and observed values.

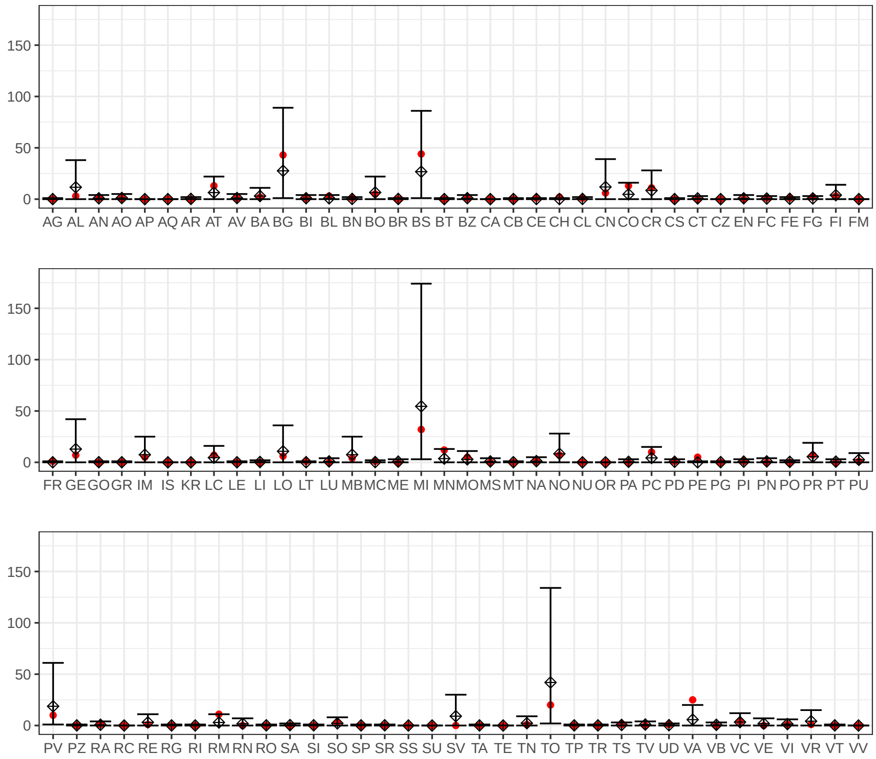

Table 2: Observed and predicted number of COVID-19 infections at 31 May 2020 according to model (1) fitted on data between 27 February 2020 and 30 May 2020 (continued).

Province	Acronym	Observed No. Infections	Predicted No. Infections
Pordenone	PN	0	0.9
Isernia	IS	0	0
Biella	BI	1	0.9
Lecco	LC	7	4.5
Lodi	LO	6	10.8
Rimini	RN	0	1.8
Prato	PO	0	0.4
Crotone	KR	0	0
Vibo Valentia	VV	0	0
Verbano-Cusio-Ossola	VB	0	0.6
Monza e della Brianza	MB	4	7.4
Fermo	FM	0	0
Barletta-Andria-Trani	BT	0	0.1
Torino	TO	20	41.9
Vercelli	VC	4	3.3
Novara	NO	7	8.3
Cuneo	CN	6	11.9
Asti	AT	13	6.4
Alessandria	AL	3	11.6
Aosta	AO	1	1.1
Imperia	IM	5	7.4
Savona	SV	0	9.1
Genova	GE	7	12.9
La Spezia	SP	0	0.1
Varese	VA	25	5.7
Como	CO	13	4.7
Sondrio	SO	3	2.1
Milano	MI	32	54.5
Bergamo	BG	43	27.6
Brescia	BS	44	26.7
Pavia	PV	10	18.7
Cremona	CR	11	8.4
Mantova	MN	12	3.7
Bolzano	BZ	1	1
Trento	TN	1	2.3
Verona	VR	1	4.2
Vicenza	VI	1	1.4
Belluno	BL	3	0.8
Treviso	TV	0	1
Venezia	VE	0	1.9
Padova	PD	1	0.6
Rovigo	RO	0	0.1
Udine	UD	1	0.4
Gorizia	GO	0	0.1
Trieste	TS	1	0.6
Piacenza	PC	10	4.3
Parma	PR	7	5.5
Reggio nell'Emilia	RE	1	3.1
Modena	MO	5	2.9
Bologna	BO	5	6.5
Ferrara	FE	1	0.4
Ravenna	RA	1	0.8
Forlì-Cesena	FC	1	0.6
Pesaro e Urbino	PU	1	2.5

Table 3: Observed and predicted number of COVID-19 infections at 31 May 2020 according to model (1) fitted on data between 27 February 2020 and 30 May 2020 (continued).

Province	Acronym	Observed No. Infections	Predicted No. Infections
Ancona	AN	1	0.8
Macerata	MC	1	0.3
Ascoli Piceno	AP	0	0
Massa Carrara	MS	0	0.9
Lucca	LU	0	0.9
Pistoia	PT	0	0.7
Firenze	FI	3	3.9
Livorno	LI	0	0.3
Pisa	PI	1	0.7
Arezzo	AR	0	0.2
Siena	SI	0	0.2
Grosseto	GR	0	0.1
Perugia	PG	0	0.2
Terni	TR	0	0.1
Viterbo	VT	0	0.1
Rieti	RI	0	0
Roma	RM	11	3
Latina	LT	1	0.1
Frosinone	FR	1	0.1
Caserta	CE	1	0.2
Benevento	BN	1	0.2
Napoli	NA	1	1.1
Avellino	AV	1	1.1
Salerno	SA	0	0.4
L'Aquila	AQ	0	0
Teramo	TE	0	0
Pescara	PE	5	0
Chieti	CH	2	0.1
Campobasso	CB	0	0.1
Foggia	FG	2	0.7
Bari	BA	2	3
Taranto	TA	0	0.1
Brindisi	BR	0	0.2
Lecce	LE	0	0.1
Potenza	PZ	0	0.1
Matera	MT	0	0.1
Cosenza	CS	0	0.1
Catanzaro	CZ	0	0
Reggio di Calabria	RC	0	0
Trapani	TP	0	0.1
Palermo	PA	0	0.5
Messina	ME	0	0.7
Agrigento	AG	0	0
Caltanissetta	CL	1	0.2
Enna	EN	0	0.8
Catania	CT	0	0.7
Ragusa	RG	0	0.1
Siracusa	SR	0	0.2
Sassari	SS	0	0
Nuoro	NU	0	0
Cagliari	CA	0	0
Oristano	OR	0	0
Sud Sardegna	SU	0	0

References

O. A. Adegboye and M. Adegboye. Spatially correlated time series and ecological niche analysis of cutaneous leishmaniasis in afghanistan. *International Journal of Environmental Research and Public Health*, 14(3), 2017.

Q. Cheng, X. Lu, J.T. Wu, Z. Liu, and J. Huang. Analysis of heterogeneous dengue transmission in guangdong in 2014 with multivariate time series model. *Scientific Reports*, 6(33755), 2016.

Leonhard Held, Michael Höhle, and Mathias Hofmann. A statistical framework for the analysis of multivariate infectious disease surveillance counts. *Statistical Modelling*, 5(3):187–199, 2005.

Ying Liu, Albert A Gayle, Annelies Wilder-Smith, and Joacim Rocklöv. The reproductive number of COVID-19 is higher compared to SARS coronavirus. *Journal of Travel Medicine*, 02 2020.

Benjamin F. Maier and Dirk Brockmann. Effective containment explains sub-exponential growth in confirmed cases of recent covid-19 outbreak in mainland china, 2020.

Sebastian Meyer and Leonhard Held. Power-law models for infectious disease spread. *The Annals of Applied Statistics*, 8(3):1612–1639, 2014.

Sebastian Meyer, Leonhard Held, and Michael Höhle. Spatio-temporal analysis of epidemic phenomena using the r package surveillance. *Journal of Statistical Software, Articles*, 77(11): 1–55, 2017.

M. Paul and L. Held. Predictive assessment of a non-linear random effects model for multivariate time series of infectious disease counts. *Statistics in Medicine*, 30(10):1118–1136, 2011.
